# Effect of cerebellar stimulation on postural control and associated resting-state functional alterations in chronic ankle instability

**DOI:** 10.3389/fspor.2026.1710598

**Published:** 2026-02-26

**Authors:** Shanshan Zheng, Yushi Chen, Zikun Wang, Yuwen Zhang, Yiran Wang, Le Yu, Weichu Tao, Qianru Li, Yang Sun, Tsz Yuen Frank Wang, Xiao'ao Xue, He Wang, Yinghui Hua

**Affiliations:** 1 Department of Sports Medicine, Huashan Hospital, Fudan University, Shanghai, China; 2 Institute of Science and Technology for Brain-inspired Intelligence, Fudan University, Shanghai, China; 3 Sports Medicine Center, Honghui Hospital, Xi'an, China; 4 Department of Orthopedics, Shanghai Clinical Research and Trial Center, Shanghai, China

**Keywords:** ankle injuries, cerebellum, chronic ankle instability, postural control, balance, transcranial direct current stimulation

## Abstract

**Background:**

Cerebellar maladaptive plasticity underlies postural deficits in chronic ankle instability (CAI), and we tested whether cerebellar-targeted transcranial direct current stimulation (tDCS) can provide a rapid, mechanism-specific postural benefit.

**Research question:**

Would a single cerebellar tDCS session yield immediate improvements in postural control along with associated alterations in cerebellar activity?

**Methods:**

In this randomized, sham-controlled laboratory study, 22 participants with CAI received active or sham cerebellar tDCS (20 min). Pre- and post-intervention assessments included: (1) laboratory-based center-of-pressure (CoP) sway during single-leg stance and clinic-based Balance Error Scoring System (BESS); (2) cerebellar regional homogeneity and amplitude of low-frequency fluctuations on resting-state functional magnetic resonance imaging.

**Results and significance:**

The tDCS group showed a significant reduction in the CoP length in the mediolateral direction (*p* = 0.020, Cohen's *d* = −0.963). However, no significant alterations were observed between the sham and tDCS groups in terms of other CoP sway or BESS outcomes. A strong association was observed between the tDCS-induced reduction in mediolateral CoP length and the improvement in activity coherence in superior lobe 3, which is involved in integrating sensory information into postural motor responses (*r* = −0.709, *p* = 0.018). Our study indicates that a single session of cerebellar tDCS can immediately alter postural control in patients with CAI and is correlated with increased coherence in the superior lobe of the cerebellum. However, the observed beneficial effects in laboratory-based CoP tests might be too subtle to be detected by clinic-based BESS outcomes.

## 1 Introduction

Lateral ankle sprains are prevalent musculoskeletal injuries that affect approximately 60% of individuals at some stage in their lives (1). Although often viewed as an innocuous injury, a significant consequence of acute sprains is their high recurrence rate and residual pathological state known as chronic ankle instability (CAI) (1). While the initial injury may cause mechanical instability, sensorimotor deficits often persist in individuals developing CAI (1, 2). Postural control impairment in CAI has garnered extensive research owing to its critical role in daily and sports activities (3–5). Effective strategies for addressing this issue are currently lacking, thereby requiring the development of more targeted, mechanism-specific interventions (6).

Central-nervous alterations likely drive CAI-related postural deficits (7) Freeman et al. (8) first proposed in 1965 that proprioceptor damage from ligament rupture distorts single-leg reflexes, and later work confirms accompanying supraspinal dysfunction (9, 10). The cerebellum plays a critical role in refining movements and maintaining stable postures (11, 12). Given the well established link between CAI and impaired postural control, researchers have long suspected lasting cerebellar involvement in this condition (13). Direct evidence, however, has only become available with advances in magnetic resonance imaging (MRI) that enable evaluation of subcortical structures (7). Using structural and resting-state MRI, Xue et al. showed that postural deficits in CAI are associated with cerebellar abnormalities, including reduced grey matter volume in the cerebellar vermis and decreased resting-state intrinsic activity in the bilateral cerebellar lobule VIIIb, as indicated by lower regional homogeneity (ReHo) (14, 15). Regrettably, the targeted interventions based on cerebellum are still lacking for CAI rehabilitation (7, 16, 17).

Transcranial direct current stimulation (tDCS) is a non-invasive technique that delivers a low scalp current to shift neuronal resting potentials, adjust regional excitability, and translate maladaptive neuroplasticity into clinical gains (18). Clinical applications of tDCS indicate its potential for improving postural stability (19). Ehsani et al. and Yosephi et al. found that anodal tDCS applied either to primary motor cortex (M1) or cerebellum improved postural stability in older adults after both a single 20-minute session and a six-session, two-week regimen, with a greater effect observed in cerebellar area (20, 21). In CAI, preliminary studies by Bruce et al. and Ma et al. employing multi-weeks of M1-based tDCS have reported improvements in dynamic balance outcomes (17, 22, 23). However, no studies have yet investigated the effects of cerebellar tDCS in CAI (17).

Accordingly, before considering a full multi-session trial, we conducted a preliminary randomized controlled study this time to evaluate the immediate effects of a single cerebellar tDCS session on postural control in patients with CAI, and we repeatedly assessed neural activity before and after stimulation to investigate potential central changes associated with any postural control improvements. We hypothesized that a 20-min cerebellar tDCS session would yield immediate improvements in postural control along with associated alterations in cerebellar activity.

## 2 Materials and methods

### 2.1 Study design

**Figure 1:**
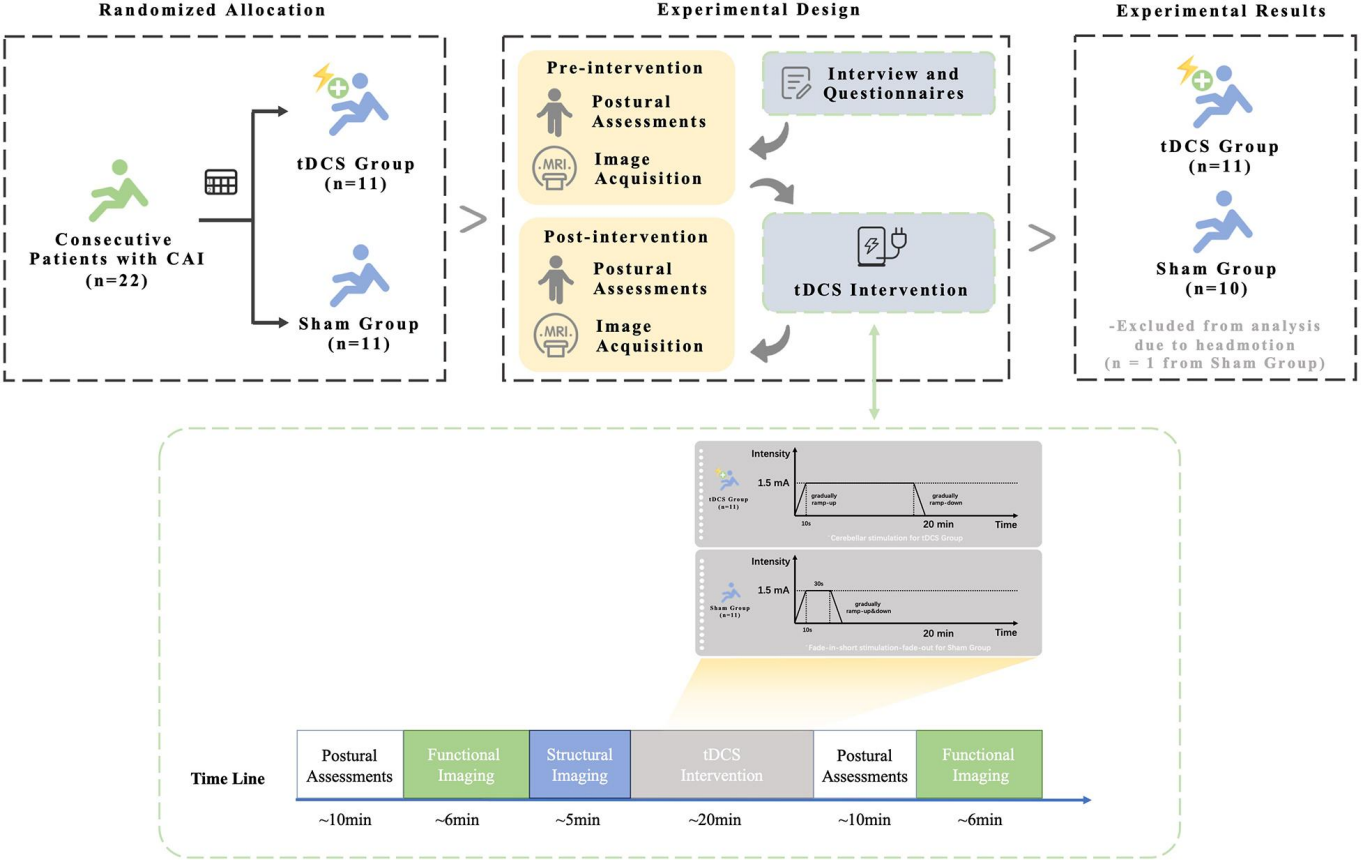
Flowchart of participant enrollment, allocation, experimental procedures, and analysis. Details of transcranial direct current stimulation (tDCS) intervention include the timeline of repeated evaluations before and after stimulation, the simulated distribution of the electrical field (ensuring direct stimulation of the cerebellar vermis and intermediate hemispheres), and the protocols of real and sham tDCS intervention.

Participants were randomly assigned to either the active tDCS or sham group and underwent postural control tests and brain MRI before and immediately after 20 min of stimulation (flowchart presented in Figure 1). Randomization was performed with a sequence generated in Microsoft Excel 2021 (Microsoft, USA) by a single investigator who was involved only in configuring the tDCS. All other researchers responsible for postural-control and MRI assessments, and for statistical analyses, were blinded to the group allocation. The study protocol was approved by the Institutional Research Ethics Committee of Huashan Hospital Affiliated with Fudan University. Informed consent was obtained from all participants before enrollment. This report adheres to the Consolidated Standards of Reporting Trials guidelines (24).

### 2.2 Participants

We calculated the *a priori* sample size in G*Power 3.1 using reported improvements in static postural control after a 20-min cerebellar tDCS session (effect size = 0.95) (20). To achieve a power of 0.8 and an *α* of 0.05, nine participants per group were required, with a 1:1 allocation ratio. To account for potential dropouts, 22 participants were recruited from the local college.

The inclusion criteria for CAI were based on the International Ankle Consortium (1): (i) a history of at least one significant ankle sprain causing pain, swelling, and disruption of normal physical activity; (ii) the first significant sprain occurring at least 12 months prior to enrollment; and (iii) a Cumberland Ankle Instability Tool (CAIT) score <24, indicating unilateral ankle instability. The exclusion criteria were (i) prior surgeries or fractures requiring realignment in the lower extremities; (ii) significant acute lower extremity injuries within the past 3 months; (iii) major medical illnesses; (iv) current use of specific medications; (v) active skin infections or latex allergies; and (vi) non-right-footedness, as determined by the preferred limb for kicking a ball, to avoid potential effects of brain lateralization on MRI analysis and interpretation.

### 2.3 Evaluation

Participants were instructed to maintain their usual sleep and physical-activity routines and to abstain from caffeine and alcohol for at least 12 h before testing. All assessments were conducted in the morning, when participants had not yet engaged in daily physical activities.

#### 2.3.1 Questionnaires

Demographic and clinical features were obtained during a brief (∼10 min) interview conducted on the experimental day, including age, sex, body mass index, Tegner scale for activity level, injured side, self-reported duration of CAI, CAIT questionnaire, Foot and Ankle Ability Measure questionnaire, numeric rating scale (NRS) for ankle pain during walking, and the total number of recurrent sprains. After the interview, participants were given the opportunity to rest in the research center until they felt fully prepared to begin the formal experiment (see Table 1).

**Table 1:** Demographics and clinical features for the group of cerebellar tDCS and sham controls.

Variable	Sham group	tDCS group	*P* value
	(*n* = 10)	(*n* = 11)	
Sex	6 female/4 male	6 female/5 male	1.000
Age (years)	25.21 ± 2.31	28.05 ± 4.88	0.111
BMI (kg/m^2^)	20.80 ± 2.11	21.91 ± 1.83	0.212
Tegner	3.00 [3.00, 4.75]	4.00 [3.50, 5.00]	0.300
Injured Side	5 Left/5 Right	5 Left/6 Right	1.000
Duration (years)	6.00 [2.00, 9.75]	10.00 [5.00, 13.50]	0.104
CAIT	16.00 [12.75, 18.75]	16.00 [14.00, 20.00]	0.377
FAAM-ADL	97.02 [93.45, 99.70]	96.43 [94.64, 98.21]	0.830
FAAM-Sports	93.75 [83.59, 96.88]	93.75 [87.50, 100.00]	0.453
NRS of pain	0.50 [0.00, 2.50]	0.00 [0.00, 1.50]	0.592
Times of Recurrent sprains	3.00 [2.25, 4.75]	3.00 [2.00, 4.00]	0.565

ADL, activities of daily living; BMI, body mass index; CAIT, Cumberland ankle instability tool; FAAM, foot and ankle ability measure; NRS, numeric rating scales; tDCS, transcranial direct current stimulation. Normally distributed continuous variable data are presented as mean ± standard deviation, and the others are presented as median [interquartile range].

#### 2.3.2 Postural control evaluation

First, postural stability was assessed with posturography on a MedTrack force plate(Xinkang Biomedical Technology, Mainland China) with a sampling frequency of 100 Hz and a cut-off frequency of 5 Hz for data filtering. Participants stood barefoot on the injured limb under eyes-open conditions and attempted to maintain balance for 15 s. This condition was selected to maximize feasibility and minimize missing pre-post data, as pilot testing showed that many CAI participants could not safely complete eyes-closed single-leg stance trials consistently due to excessive sway. The stance required the weight bearing knee to be slightly flexed, the non-weight bearing hip and knee slightly flexed, and the hands placed on the iliac crests. We chose 15s because CAI patients have reduced stability and 10–20 s is the most common duration in this population (25, 26). The key variables analyzed were traditional linear center-of-pressure (CoP) measures: average displacement length of the CoP trajectory (total, mediolateral, and anteroposterior) and the 95% confidence-ellipse area. Higher CoP lengths and areas indicate greater postural-control deficit. Each participant completed three trials separated by 30 s, and the mean value was used for analysis.

In addition, eyes-closed postural control was assessed using the Balance Error Scoring System (BESS). This test comprises three 20 s stances with eyes closed, double-leg (feet together), single-leg (injured side), and tandem (injured foot directly behind the contralateral heel), performed on both firm and foam surfaces, for six conditions in total. Errors were defined as any deviation from the stance (opening eyes, lifting hands off hips, stepping, stumbling or falling, lifting forefoot or heel, abducting the hip by >30°, or failing to return to the test position within 5 s). A maximum of 10 errors was recorded per trial, and the total error count across all six conditions was recorded, with higher scores indicating poorer postural control. Each participant performed the BESS once, mirroring routine clinical practice.

To minimize participant fatigue, while ensuring measurements were completed within the optimal post-stimulation window of tDCS, we decided not to include dynamic balance tasks in this study.

#### 2.3.3 MRI data acquisition and analysis

To assess the central effects of cerebellar tDCS, resting-state functional MRI (rs-fMRI) was performed using a 3.0 Tesla PRISMA scanner (Siemens, Erlangen, Germany). Analysis of rs-fMRI data used the RESTplus version 1.21 (27). MRI preprocessing included slice-timing and head-motion correction, co-registration, unified segmentation, spatial normalization, detrending of thermal drift, and regression of nuisance covariates (27). Participants with a mean frame-wise displacement of head motion larger than 0.2 mm were excluded. The fractional amplitude of low-frequency fluctuation (fALFF) and regional homogeneity (ReHo) were used to evaluate regional intrinsic activity intensity and coherence, respectively (28). More details on MRI data acquisition and preprocessing are found in Supplementary Digital Content S1. Because the goal of the neuro-imaging component was to link brain changes to the observed postural benefits of tDCS, correlation analyses were limited to rs-fMRI measures that exhibited significant between-group differences in pre- to post-intervention change.

### 2.4 Cerebellar TDCS stimulation

Cerebellar tDCS was administered using an ActivaDose II device. The anode and cathode electrodes were positioned within two 5 cm ×  5 cm sponges soaked in 14 mL of saline solution. The anode was placed over approximately 1 cm below the inion of occipital bone, and the cathode on the middle portion of right deltoid (20). The electrode arrangement was verified using finite element analysis (20, 29) (see Figure 1). In the tDCS group, participants received 1.5 mA anodal stimulation for 20 min, which was gradually adjusted during the initial and final 10 s to minimize side effects (22). The sham group underwent a fade-in-short-stimulation-fade-out protocol in which stimulation was turned off after 30 s (see Figure 1) (20, 21, 30) During the intervention, subjects sat in suitable chairs and avoided unnecessary physical and mental activity. After the intervention, participants' guesses of tDCS intervention allocation and a side-effects NRS was administered. ﻿(See Supplementary Digital Content S2).

### 2.5 Statistical analyses

Data analysis was performed using R (version 4.1.2). Normality was checked with Kolmogorov–Smirnov: demographics were normal, but questionnaire, postural control, and rs-fMRI data were not. Baseline characteristics and blinding were compared with t- or chi-square tests; non-normal baselines and tDCS side effects used Mann–Whitney U. tDCS efficacy was assessed by Mann–Whitney U on change scores (post- minus pre-intervention) for postural control and rs-fMRI. Cohen's d (95% confidence intervals) quantified between-group effects for postural control and rs-fMRI (small 0.2–0.5, moderate 0.5–0.8, large >0.8). Spearman tests examined correlations between significant postural control and rs-fMRI changes within the tDCS group. Significance was set at two-tailed *p* < 0.05. Because this was an exploratory preliminary study, no multiple-comparison corrections were applied; only rs-fMRI outcomes that correlated with postural measures were considered meaningful.

## 3 Results

Of 22 volunteers, one control participant was excluded for excessive head motion on the initial fMRI and withdrew, received no tDCS, and his pre-intervention CoP and BESS data were omitted from analysis. Figure 1 presents the flowchart of participant enrolment, group allocation, experimental procedures, and data analysis. The final sample consisted of 10 patients in the sham group and 11 in the active tDCS group. Groups were comparable at baseline: identical sex distribution and no differences in age, BMI, Tegner level, injury duration, CAIT, FAAM, or recurrent-sprain counts (see Table 1). Stimulation was well tolerated; only initial tingling was higher in the sham group (*p* = 0.024) (see Supplementary Digital Content S2). Blinding remained intact, with guess accuracy no better than chance (sham group: active = 2; sham = 3; unsure = 5) and tDCS (tDCS group: active = 3; sham = 5; unsure = 3) (chi-square = 1.155; df = 2; *p* = 0.561).

**Figure 2:**
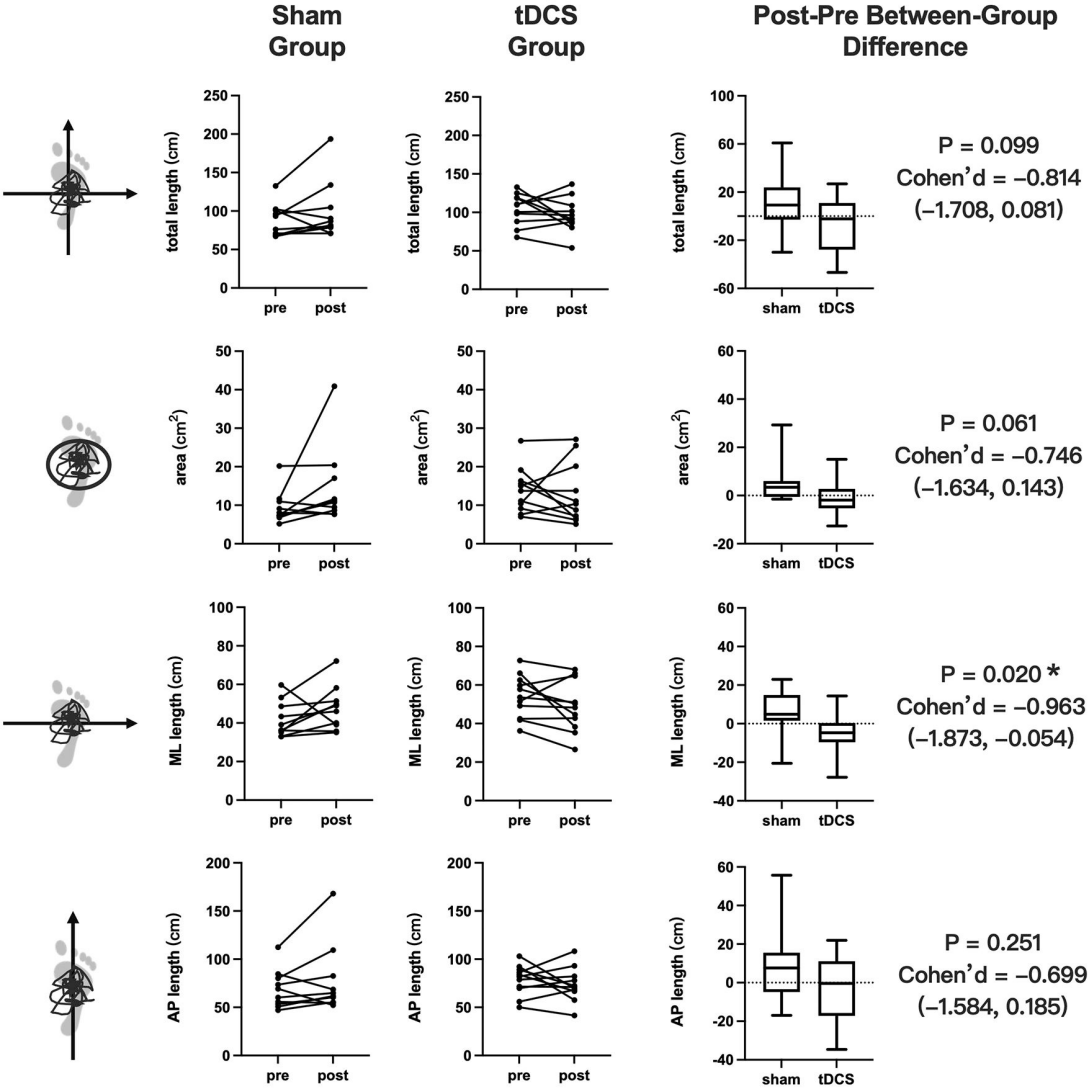
Pre- and post-intervention and between-group comparisons of postural control outcomes. ML, mediolateral; AP, anteroposterior; tDCS, transcranial direct current stimulation. An asterisk (*) denotes a statistically significant (*p* < 0.05) difference between the sham and tDCS groups in the magnitude of change from pre- to post-intervention.

**Figure 3:**
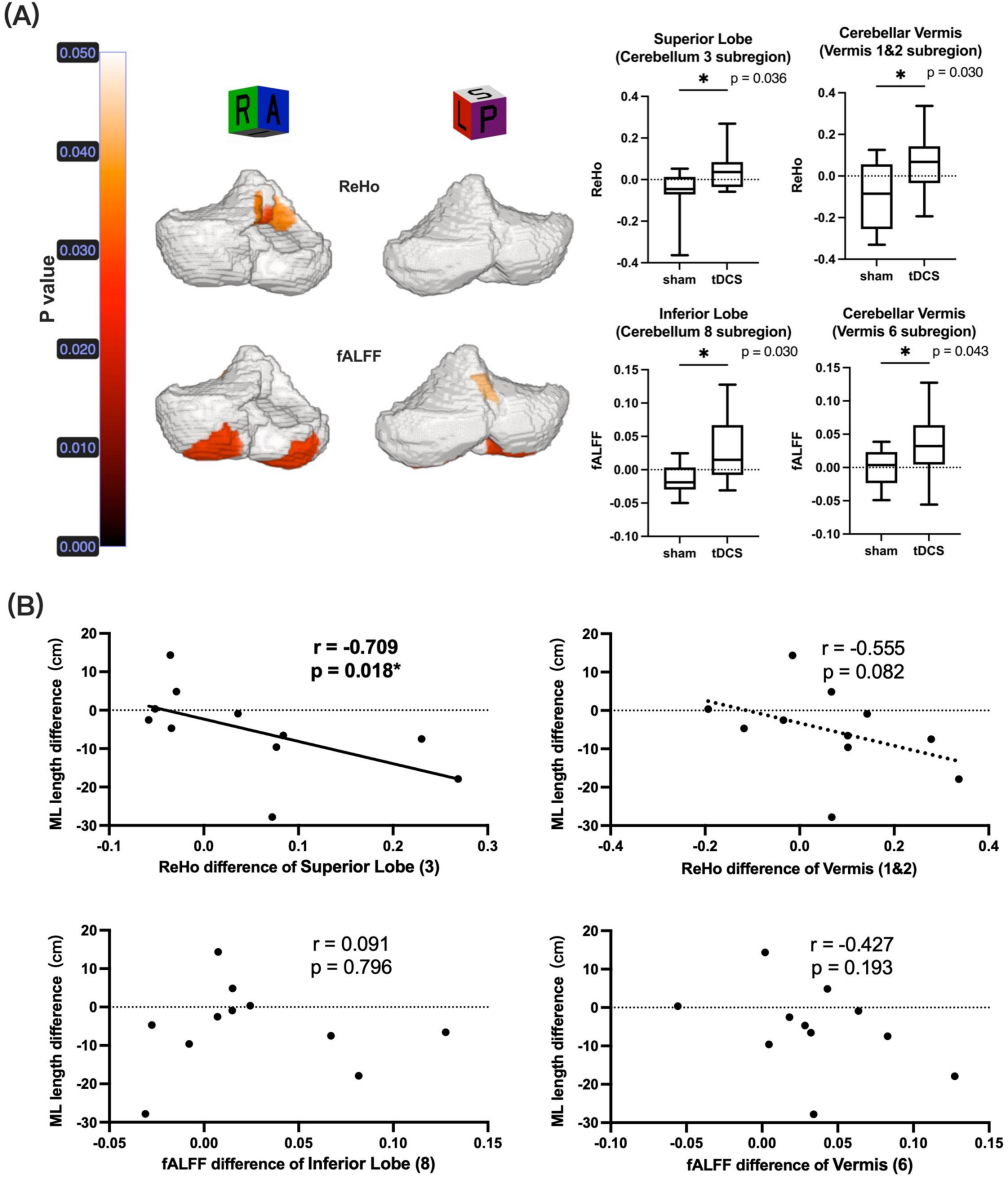
Schematic of cerebellar activity with significant between-group differences in pre- and postintervention changes (A, highlighted in orange-red). Scatter plot of the relationship between altered cerebellar activity and postural control outcomes within the tDCS group **(B)** ML, mediolateral; tDCS, transcranial direct current stimulation; fALFF, fractional amplitude of low-frequency fluctuation; ReHo, regional homogeneity. An asterisk (*) indicates a statistically significant correlation (*p* < 0.05).

Instrumented laboratory-based CoP scores revealed increased sway in the sham group and decreased sway in the tDCS group across various parameters (see Figure 2). In the sham group, increased sway was observed in the total [9.29 (1.06, 17.43) cm], mediolateral [4.86 (2.35, 12.62) cm], and anteroposterior [7.67 (0.22, 10.50) cm] directions of the CoP length trajectory, and in the CoP area [3.45 (−0.18, 4.59) cm^2^]. In the tDCS group, decreased sway was observed in the median total [−2.17 (−22.03, 7.21) cm], mediolateral [−4.68 (−8.54, −0.28) cm], and anteroposterior [−0.41 (−16.91, 9.60) cm] directions of the CoP length trajectory and median CoP area [−1.92 (−4.59, 1.55) cm^2^]. In comparison, the tDCS group showed a significant improvement with a large effect size in the median mediolateral direction of the CoP length trajectory (*p* = 0.020; Cohen's d = −0.963; 95% CI: −1.873, −0.054). However, no significant differences were observed between the sham [−4.00 (−7.00, −1.00) errors] and tDCS [−1.00 (−3.00, 0.00) errors] groups in noninstrumented clinic-based BESS scores (*p* = 0.158) (see Table 2). In the tDCS group, ReHo rose in vermis 1–2 (*p* = 0.030; *d* = 0.918, 0.013–1.822) and superior lobe 3 (*p* = 0.036; d = 1.133, 0.204–2.062), while fALFF increased in vermis 6 (*p* = 0.043; d = 0.952, 0.044–1.860) and inferior lobe 8 (*p* = 0.030; d = 1.233, 0.291–2.174), all with large effect sizes. ReHo improvement in superior lobe 3 correlated strongly with the tDCS-related drop in mediolateral CoP length in the tDCS group (r = −0.709, *p* = 0.018) (see Supplementary Digital Content S3 and Figure 3).

**Table 2:** Pre- and post-intervention and between-group comparisons of balance error scoring system scores.

BESS condition	Sham (*n* = 10)		tDCS (*n* = 11)		*P* value	Cohen's d
	Pre	Post	Pre	Post		
Double-legged firm surface	0 [0,0]	0 [0,0]	0 [0,0]	0 [0,0]	1.000	Am
Single-legged firm surface	0 [0,3.5]	0 [0,2.5]	0 [0,2]	0 [0,2]	0.992	−0.098 (−0.955, 0.758)
Tandem firm surface	1 [1,3]	0 [0,2.25]	0 [0,1]	0 [0,1]	0.140	−0.608 (−1.486, 0.270)
Double-legged foam surface	0 [0,0.25]	0 [0,0]	0 [0,0]	0 [0,0]	0.214	-
Single-legged foam surface	5 [5,8.5]	2.75 [2.75,7.75]	4 [4,10]	3 [3,6]	0.120	−0.442 (−1.309, 0.426)
Tandem foam surface	3.75 [3.75,7.75]	3 [3,5.5]	2 [2,5]	2 [2,4]	0.636	−0.236 (−1.096, 0.624)
Total Score	13 [13,20.25]	6.5 [6.5,18]	9 [9,13]	7 [7,12]	0.159	−0.682 (−1.565, 0.202)

Scores are reported as median [inter-quartile range]. Higher error scores indicating greater postural control deficits. The efficacy of tDCS was evaluated using the Mann–Whitney U test to compare score changes (post-intervention minus pre-intervention) in postural control outcomes between groups.

## 4 Discussion

The most significant outcome of this exploratory study was that a single cerebellar tDCS session improved single-leg static postural control and enhanced coherence in the cerebellar superior lobe vs. sham. This preliminary study is the first to apply cerebellar tDCS to CAI.

### 4.1 Impact of cerebellar TDCS on postural control in CAI

The cerebellum receives ankle proprioceptive input through spinocerebellar tracts and interfaces widely with sensorimotor cortex, underpinning movement control and postural stability (11, 12). After an acute ankle sprain, early physiotherapy appropriately focuses on the rapid reduction of pain and swelling. Once chronic ankle instability develops, however, rehabilitation priorities must shift toward correcting persistent sensorimotor deficits, particularly proprioceptive deafferentation and the maladaptive cortical–cerebellar reorganization that accompanies it (14, 15, 31). Even when chronic stage programmes include neuromuscular retraining, they may not fully reverse these deeply embedded neuroplastic changes, which can temper the overall effectiveness of conventional therapy (16, 32). Although cerebellar tDCS may not directly influence ankle deafferentation, we speculate that it could modulate cerebellar processing and cerebello–cortical functional connectivity (20, 21), thereby facilitating sensory integration (e.g., sensory reweighting) and improving the utilization of remaining proprioceptive inputs during postural control.Further basic research is needed to elucidate the physiological cascades activated by cerebellar tDCS in CAI.

CoP sway length rose in the sham group, likely from fatigue caused by repeat testing and MRI, whereas the tDCS group maintained or improved stability, mirroring earlier tDCS results in other neurologic cohorts (20). This immediate effect, however, may not translate into meaningful clinical gains. Because people with CAI show greater sway in both mediolateral and anteroposterior directions than uninjured controls (26) intervention chiefly reduced mediolateral sway, the postural correction appears only partial. Restricting the hands to the iliac crests likely made mediolateral stabilization more challenging, rendering mediolateral CoP a more sensitive indicator of tDCS effects (33, 34). Also, because lateral ligament injury and hip abductor weakness in CAI chiefly impair mediolateral control, improvements may be most evident in that direction (35). However, the clinical meaningfulness of these changes is difficult to determine because MCID (or universal clinical cutoffs) for CoP measures in CAI have not been established. Therefore, the observed reduction in mediolateral CoP length should be interpreted as a laboratory level, direction specific improvement rather than definitive evidence of patient perceived benefit.

Furthermore, our use of non-instrumented clinic-based BESS evaluations did not reveal significant differences between the active and sham tDCS groups. The BESS is popular in CAI balance assessments because it does not require expensive forceplate equipment, which further includes double-leg, single-leg, and tandem stances on both firm and foam surfaces, providing a more comprehensive view of postural control than a single-leg stance on a firm plate (36). Although the relatively lacked sensitivity of the non-instrumented clinic-based BESS evaluations than the CoP test, the conflicting findings between the CoP outcomes and the BESS scores after tDCS might also implying that the improvements that observed in experimental conditions may be too subtle for the unsensitive clinical observation, also the limited functional transfer of tDCS-induced effects (36). Therefore, the clinical significance of the observed postural sway alternations should be interpreted cautiously, and we therefore emphasize the exploratory nature of this laboratory-based study (37).

Anodal cerebellar tDCS is thought to depolarize Purkinje cells, reduce cerebellar-to-cortex inhibition and enhance dentato-thalamo-cortical drive-mechanisms supported by rodent slice studies and paired-pulse transcranial magnetic stimulation in humans (38, 39). Converging rs-fMRI evidence further suggests that tDCS may modulate ReHo, a marker of local resting-state functional connectivity (40, 41). A single 20 min session already trims sway and speeds postural adaptation in older individuals, but ours is the first to examine this pathway in CAI (20). We found cerebellar tDCS-induced gains in regional homogeneity within lobule 3/vermis 1–2 hubs that integrate ankle afferents, accompanied by reduced mediolateral CoP sway, consistent with the superior lobe's role in refining sensorimotor responses under unstable conditions (42). Functionally, increased ReHo may indicate enhanced local resting-state functional integration, potentially supporting more efficient sensory to motor updating for postural control.

By comparison, M1 tDCS in CAI mainly heightens corticospinal excitability and improves dynamic tasks such as hopping or landing, without directly targeting sensory prediction (17, 22, 23). Thus, cerebellar and M1 tDCS likely act at complementary stages: cerebellar tDCS sharpens sensory integration, whereas M1 tDCS amplifies motor execution. Future testing combined or sequential stimulation could reveal additive clinical benefits in CAI.

### 4.2 Clinical implications

These findings highlight the urgent need for mechanism-specific CAI rehabilitation, because strength, range of motion and balance only programs are often insufficient (16). Ankle sprain and CAI patients also show brain structural and functional adaptations vs. healthy controls or copers, and these changes correlate with clinical outcomes and can drive persistent dysfunction. Adding targeted strategies that enhance central adaptation to standard training could therefore broaden effective neuroadaptive treatment options for CAI (7). Nevertheless, routine cerebellar tDCS cannot yet be advised; larger, longer, robust, multicenter trials are required to confirm clinically meaningful effects in diverse populations and to assess patient acceptance. Clinicians should provide clear educational resources on neuroadaptive techniques to foster sustained compliance.

### 4.3 Limitations

Our findings should be interpreted in light of the limitations of the study. First, the sample size might be inadequate for assessing certain effects of cerebellar tDCS. Also, omitting strict multiple-comparison corrections also substantially increases the risk of Type I errors and may inflate the likelihood of false-positive findings; the uncorrected rs-fMRI results should therefore be interpreted with caution. Second, we employed conventional tDCS, which, despite its wide use and feasibility for home application, has limited spatial precision and may activate adjacent neural structures due to relatively large electrodes and diffuse electric fields. In addition, the use of a 5 × 5 cm midline anodal electrode and a stimulation intensity of 1.5 mA, although consistent with prior CAI studies, is lower than that used in many tDCS protocols and does not ensure strict confinement of stimulation to the cerebellar vermis (20, 21). Third, although CoP outcomes during single-leg stance are widely used in CAI studies, they may miss a substantial proportion of whole-body center of mass acceleration, potentially reducing the sensitivity of our evaluation (43, 44). In addition, instrumented CoP testing was performed under eyes open conditions only, as pilot testing indicated that many CAI participants were unable to complete eyes closed trials reliably, which may have limited the sensitivity to detect balance deficits under visual deprivation. Furthermore, matched biomechanical assessments during the BESS tasks and statistical parametric mapping of the CoP time profiles were not performed because of time constraints and limited research resources. Fourth, the physiological interpretation of rs-fMRI is less clear than that of task-based fMRI, and unmonitored cognitive activity during scanning could bias the results. Fifth, despite efforts to minimize the time gap between stimulation and evaluation, bias could still be introduced, especially when correlating behavioral tests with MRI results. Sixth, our study procedures, including the demographic and clinical interview, tDCS stimulation, and repeated postural and MRI evaluations, could have induced participant fatigue and thereby influenced the results. Seventh, learning effects resulting from repeated postural testing might bias the interpretation of the alterations produced by tDCS. Eighth, methodological consideration should be noted regarding the testing posture. Allowing slight knee flexion, which was adopted based on previous evidence, may potentially constrain anterior–posterior sway. Future studies should further examine the effects of knee and ankle joint positioning on directional postural sway. Finally, our study did not exclude the presence of mechanical laxity in participants with CAI, which could obscure functional improvements due to tDCS.

## 5 Conclusion

Our study demonstrated that a single session of cerebellar tDCS enhanced postural control in patients with CAI and was correlated with increased coherence in the superior lobe of the cerebellum. However, the observed effects are subtle, which indicates the need for further research involving more cerebellar tDCS sessions and combined exercise therapy to amplify the observed benefits.

## Data Availability

The raw data supporting the conclusions of this article will be made available by the authors, without undue reservation.
