# Supplementary material for: Effect of cerebellar stimulation on postural control and associated resting-state functional alterations in chronic ankle instability
Source: Front Sports Act Living. 2026 Feb 26;8:1710598. doi: 10.3389/fspor.2026.1710598 (PMC12979079; doi:10.3389/fspor.2026.1710598)
Supplement: Supplementary file 1 [file Table1.docx]

**Supplemental Digital Content 1. Details of MRI data acquisition and analysis**

**Supplemental Digital Content 1.1. Details of MRI data acquisition**

A 32-channel head coil was equipped in the scanner. The participants were fixed with their heads cushioned by foam pads to minimize head motion. Two appropriately sized earplugs were applied to reduce noise, and the scanning would be terminated if the subject complained of discomfort through a handheld alarm. Before the functional session, the subjects were asked to stay awake and focus on the cross on the screen without actively moving or thinking, which is a part of the requirement written in the informed consent and signed by the participants as the guarantee to follow the researcher’s command during the test. The scanning sequence was as follows: (i) structural imaging of ﻿T1-weighted Magnetization-Prepared Rapid Gradient-Echo ﻿sequence, matrix size = 208 * 300 * 320 slices, 0.8 mm^3^ isotropic voxels, slice gap = 0 mm, repetition time = 2500 ms, echo time = 2.22 ms, flip angle = 8°, (ii) functional imaging of ﻿echo-planar imaging sequence, matrix size = 104 * 104 * 72 slices, 2.0 mm^3^ isotropic voxels, slice gap = 0 mm, repetition time = 800 ms, echo time = 37 ms, flip angle = 52°, number of acquisitions = 460, ascending interleaved acquisition of axial slices.

**Supplemental Digital Content 1.2. Details of rs-fMRI data analysis**

The analyses for the rs-fMRI were performed using the Resting-State fMRI Data Analysis Toolkit (RESTplus) version 1.21 ^1^.

For the functional images, the following features were considered in each image: (i) the first 10 time points of functional images were discarded for stabilization of the magnetic field, (ii) slice-time correction (referred to the middle slice of each run) was applied to correct acquisition delay between slices, (iii) head motion correction was applied by realigning the functional images to the middle image, with the mean functional image produced, (iv) structural images were co-registered to the mean functional image by rigid body transformation, (v) the co-registered structural images were segments into grey matter, white matter and cerebrospinal fluid components using unified segmentation, (vi) segmented images were used to compute transformation parameters and then the motion-corrected functional imaged were spatially normalized to MNI space, (vii) thermal drift during functional scanning was corrected by detrending, (viii) nuisance covariates, including linear trend, the 24 parameters of head motions of Friston (six head motion parameters, six head motion parameters one time point before, and the 12 corresponding squared items), signal noise of white matter and cerebrospinal fluid were regressed out from the functional images.

(ix-1) Computation of fALFF: time series for each voxel that was transformed to the frequency domain using a fast Fourier transformation. Average amplitudes within the low-frequency band (0.01–0.08 Hz) were divided by those across the entire frequency range (0.01–0.27 Hz) and then forms a fALFF map that standardized by the whole brain mean value for each subject ^2^.

(ix-2) Computation of ReHo: After additionally appling band-pass temporal filter (0.01–0.08 Hz), Kendall’s coefficient concordance were calculated based on the similarity of the time series of a single voxel to its nearest 27 neighbors, and then forms a ReHo map that standardized by the whole brain mean value for each subject ^3^.

The individual maps of fALFF and ReHo were then divided by region of interest analysis, which estimates the averaged values of the outcome map within all included voxels of the cerebellar regions within automated anatomical labeling atlas.^4,5^

**Reference:**

1. Jia XZ, Wang J, Sun HY, et al. RESTplus: an improved toolkit for resting-state functional magnetic resonance imaging data processing. *Sci Bull*. 2019;64(14):953-954. doi:10.1016/j.scib.2019.05.008

2. Zou QH, Zhu CZ, Yang Y, et al. An improved approach to detection of amplitude of low-frequency fluctuation (ALFF) for resting-state fMRI: Fractional ALFF. *J Neurosci Methods*. 2008;172(1):137-141. doi:10.1016/j.jneumeth.2008.04.012

3. Zang Y, Jiang T, Lu Y, He Y, Tian L. Regional homogeneity approach to fMRI data analysis. *Neuroimage*. 2004;22(1):394-400. doi:10.1016/j.neuroimage.2003.12.030

4. Tzourio-Mazoyer N, Landeau B, Papathanassiou D, et al. Automated anatomical labeling of activations in SPM using a macroscopic anatomical parcellation of the MNI MRI single-subject brain. *Neuroimage*. 2002;15(1):273-289. doi:10.1006/nimg.2001.0978

5. Xue X, Zhang Y, Li S, Xu H, Chen S, Hua Y. Lateral ankle instability-induced neuroplasticity in brain grey matter: A voxel-based morphometry MRI study. *J Sci Med Sport*. 2021;24(12):1240-1244. doi:10.1016/j.jsams.2021.06.013
