# Supplementary material for: Effect of cerebellar stimulation on postural control and associated resting-state functional alterations in chronic ankle instability
Source: Front Sports Act Living. 2026 Feb 26;8:1710598. doi: 10.3389/fspor.2026.1710598 (PMC12979079; doi:10.3389/fspor.2026.1710598)
Supplement: Supplementary file 2 [file Table2.docx]

**Supplemental Digital Content 2. Evaluation of side-effects during tDCS intervention through NRS.**

.

|  |  | Sham  （n=10） | tDCS  （n=11） | P value |
| --- | --- | --- | --- | --- |
| ﻿Tingling sensation | ﻿Beginning | 1.00 (0-7) | 0.00 (0-3) | 0.024 |
|  | ﻿Middle | 1.00 (0-3) | 0.00 (0-3) | 0.124 |
|  | ﻿End | 1.00 (0-3) | 0.00 (0-3) | 0.673 |
| ﻿Itching sensation | ﻿Beginning | 0.00 (0-1) | 0.00 (0-1) | 0.326 |
|  | ﻿Middle | 0.00 (0-1) | 0.00 (0-3) | 0.303 |
|  | ﻿End | 0.00 (0-1) | 0.00 (0-3) | 0.303 |
| ﻿Burning sensation | ﻿Beginning | 0.00 (0-1) | 0.00 (0-3) | 1.000 |
|  | ﻿Middle | 0.00 (0-2) | 0.00 (0-3) | 0.113 |
|  | ﻿End | 0.00 (0-1) | 0.00 (0-3) | 0.918 |
| ﻿Pain | ﻿Beginning | 0.00 (0-3) | 0.00 (0-3) | 0.844 |
|  | ﻿Middle | 0.00 (0-1) | 0.00 (0-3) | 0.741 |
|  | ﻿End | 0.00 (0-1) | 0.00 (0-3) | 0.704 |
| ﻿﻿Headache | ﻿Beginning | 0.00 (0-1) | 0.00 (0-3) | 0.563 |
|  | ﻿Middle | 0.00 (0-1) | 0.00 (0-3) | 0.636 |
|  | ﻿End | 0.00 (0-2) | 0.00 (0-3) | 0.563 |

﻿Numeric Rating Scale (NRS): 0 is rated as no sensation and 10 rated as the worst sensation imaginable. Scores are reported as median (range).
