# Supplementary material for: Effect of cerebellar stimulation on postural control and associated resting-state functional alterations in chronic ankle instability
Source: Front Sports Act Living. 2026 Feb 26;8:1710598. doi: 10.3389/fspor.2026.1710598 (PMC12979079; doi:10.3389/fspor.2026.1710598)
Supplement: Supplementary file 3 [file Table3.docx]

**Supplemental Digital Content 3. Between-group comparison of the change scores (post-intervention minus pre-intervention) in cerebellar rs-fMRI outcomes**

|  | tDCS Group | | | Sham Group | | |  |  |
| --- | --- | --- | --- | --- | --- | --- | --- | --- |
|  | Median | IQR- | IQR+ | Median | IQR- | IQR+ | P value | Cohen’s d (95% CI) |
| fALFF |  |  |  |  |  |  |  |  |
| Crus1 | 0.020 | -0.005 | 0.032 | 0.018 | -0.028 | 0.043 | 0.863 | 0.048 (-0.808, 0.905) |
| Crus2 | 0.011 | -0.003 | 0.079 | -0.007 | -0.034 | 0.031 | 0.314 | 0.326 (-0.536, 1.189) |
| Superior_3 | -0.016 | -0.034 | 0.027 | -0.027 | -0.102 | 0.016 | 0.512 | 0.161 (-0.697, 1.018) |
| Superior_4_5 | -0.000 | -0.027 | 0.040 | -0.001 | -0.018 | 0.014 | 0.918 | 0.025 (-0.831, 0.882) |
| Superior_6 | 0.006 | -0.005 | 0.046 | -0.010 | -0.029 | 0.008 | 0.282 | 0.481 (-0.389, 1.351) |
| Inferior_7 | 0.008 | -0.010 | 0.071 | -0.008 | -0.033 | 0.020 | 0.223 | 0.312 (-0.550, 1.174) |
| Inferior_8 | **0.015** | **-0.000** | **0.046** | **-0.019** | **-0.025** | **-0.001** | **0.029** | **1.233 (0.291, 2.174)** |
| Inferior_9 | -0.003 | -0.013 | 0.023 | -0.024 | -0.063 | 0.010 | 0.314 | 0.501 (-0.370, 1.372) |
| Inferior_10 | -0.015 | -0.023 | 0.028 | -0.020 | -0.063 | -0.004 | 0.282 | 0.123 (-0.734, 0.980) |
| Vermis_1_2 | 0.002 | -0.055 | 0.023 | 0.013 | -0.110 | 0.084 | 0.863 | -0.102 (-0.959, 0.755) |
| Vermis_3 | -0.004 | -0.027 | 0.030 | -0.004 | -0.105 | 0.026 | 0.756 | 0.000 (-0.856, 0.856) |
| Vermis_4_5 | -0.013 | -0.021 | 0.036 | -0.002 | -0.029 | 0.039 | 1.000 | -0.238 (-1.097, 0.622) |
| Vermis_6 | **0.032** | **0.011** | **0.053** | **0.004** | **-0.018** | **0.019** | **0.043** | **0.952 (0.044, 1.860)** |
| Vermis_7 | 0.026 | -0.017 | 0.057 | 0.002 | -0.007 | 0.023 | 0.468 | 0.563 (-0.312, 1.438) |
| Vermis_8 | -0.001 | -0.025 | 0.038 | -0.016 | -0.044 | -0.002 | 0.173 | 0.374 (-0.490, 1.239) |
| Vermis_9 | 0.003 | -0.044 | 0.030 | -0.007 | -0.051 | 0.018 | 0.705 | 0.188 (-0.670, 1.047) |
| Vermis_10 | -0.006 | -0.062 | 0.036 | -0.022 | -0.101 | 0.027 | 0.705 | 0.191 (-0.668, 1.049) |
| ReHo |  |  |  |  |  |  |  |  |
| Crus1 | 0.008 | -0.069 | 0.070 | 0.003 | -0.127 | 0.120 | 0.863 | 0.034 (-0.822, 0.891) |
| Crus2 | 0.002 | -0.060 | 0.123 | -0.009 | -0.079 | 0.091 | 0.705 | 0.084 (-0.773, 0.941) |
| Superior_3 | **0.036** | **-0.035** | **0.080** | **-0.046** | **-0.067** | **0.007** | **0.036** | **1.133 (0.204, 2.062)** |
| Superior_4_5 | 0.008 | -0.025 | 0.132 | -0.003 | -0.046 | 0.037 | 0.512 | 0.117 (-0.741, 0.974) |
| Superior_6 | -0.029 | -0.131 | 0.081 | 0.002 | -0.072 | 0.033 | 1.000 | -0.246 (-1.106, 0.614) |
| Inferior_7 | 0.045 | -0.076 | 0.082 | -0.034 | -0.055 | 0.041 | 0.349 | 0.806 (-0.088, 1.700) |
| Inferior_8 | 0.003 | -0.048 | 0.146 | -0.006 | -0.082 | 0.017 | 0.282 | 0.078 (-0.779, 0.934) |
| Inferior_9 | 0.006 | -0.039 | 0.139 | -0.037 | -0.052 | -0.007 | 0.132 | 0.437 (-0.430, 1.305) |
| Inferior_10 | 0.028 | -0.076 | 0.147 | -0.014 | -0.030 | 0.005 | 0.387 | 0.347 (-0.517, 1.210) |
| Vermis_1_2 | **0.068** | **-0.025** | **0.122** | **-0.085** | **-0.235** | **0.053** | **0.029** | **0.918 (0.013, 1.822)** |
| Vermis_3 | 0.048 | -0.035 | 0.139 | -0.032 | -0.073 | 0.005 | 0.152 | 0.787 (-0.105, 1.680) |
| Vermis_4_5 | 0.011 | -0.037 | 0.079 | -0.003 | -0.034 | 0.094 | 0.918 | 0.155 (-0.703, 1.013) |
| Vermis_6 | 0.005 | -0.134 | 0.192 | 0.027 | -0.062 | 0.081 | 0.973 | -0.116 (-0.973, 0.741) |
| Vermis_7 | 0.008 | -0.029 | 0.116 | -0.046 | -0.066 | 0.038 | 0.282 | 0.573 (-0.303, 1.448) |
| Vermis_8 | -0.019 | -0.074 | 0.069 | -0.018 | -0.096 | 0.050 | 0.705 | -0.009 (-0.866, 0.847) |
| Vermis_9 | 0.064 | -0.057 | 0.127 | -0.016 | -0.166 | 0.055 | 0.349 | 0.534 (-0.339, 1.407) |
| Vermis_10 | 0.020 | -0.047 | 0.171 | 0.006 | -0.055 | 0.046 | 0.314 | 0.109 (-0.748, 0.966) |

fALFF, fractional amplitude of low-frequency fluctuation; ReHo, regional homogeneity; IQR, interquartile range; rs-fMRI, resting-state functional magnetic resonance imaging; CI, confidence interval.
